# *Clostridioides difficile* Colonization Is Differentially Associated With Gut Microbiome Profiles by Infant Feeding Modality at 3–4 Months of Age

**DOI:** 10.3389/fimmu.2019.02866

**Published:** 2019-12-11

**Authors:** Kelsea M. Drall, Hein M. Tun, Nadia P. Morales-Lizcano, Theodore B. Konya, David S. Guttman, Catherine J. Field, Rupasri Mandal, David S. Wishart, Allan B. Becker, Meghan B. Azad, Diana L. Lefebvre, Piush J. Mandhane, Theo J. Moraes, Malcolm R. Sears, Stuart E. Turvey, Padmaja Subbarao, James A. Scott, Anita L. Kozyrskyj

**Affiliations:** ^1^Department of Pediatrics, University of Alberta, Edmonton, AB, Canada; ^2^Department of Cell & Systems Biology, University of Toronto, Toronto, ON, Canada; ^3^Dalla Lana School of Public Health, University of Toronto, Toronto, ON, Canada; ^4^Centre for the Analysis of Genome Evolution & Function, University of Toronto, Toronto, ON, Canada; ^5^Department of Agricultural, Food and Nutritional Science, University of Alberta, Edmonton, AB, Canada; ^6^Department of Biological Sciences, University of Alberta, Edmonton, AB, Canada; ^7^Department of Pediatrics and Child Health, Children's Hospital Research Institute of Manitoba, University of Manitoba, Winnipeg, MB, Canada; ^8^Department of Medicine, McMaster University, Hamilton, ON, Canada; ^9^Department of Pediatrics, Hospital for Sick Children, University of Toronto, Toronto, ON, Canada; ^10^Department of Pediatrics, BC Children's Hospital, University of British Columbia, Vancouver, BC, Canada

**Keywords:** *Clostridioides difficile*, sIgA, SCFA, infant feeding, microbiome, gut microbiota, metabolites

## Abstract

Colonization with *Clostridioides difficile* occurs in up to half of infants under the age of 3 months, is strongly influenced by feeding modality and is largely asymptomatic. In spite of this, *C. difficile*'s presence has been associated with susceptibility to chronic disease later in childhood, perhaps by promoting or benefiting from changes in infant gut microbiome development, including colonization with pathogenic bacteria and disrupted production of microbial bioactive metabolites and proteins. In this study, the microbiomes of 1554 infants from the CHILD Cohort Study were described according to *C. difficile* colonization status and feeding mode at 3–4 months of age. *C. difficile* colonization was associated with a different gut microbiome profile in exclusively breastfed (EBF) vs. exclusively formula fed (EFF) infants. EBF infants colonized with *C. difficile* had an increased relative abundance of Firmicutes and Proteobacteria, decreased relative abundance of Bifidobacteriaceae, greater microbiota alpha-diversity, greater detectable fecal short chain fatty acids (SCFA), and lower detectable fecal secretory Immunoglobulin A (sIgA) than those not colonized. Similar but less pronounced differences were seen among partially breastfed infants (PBF) but EFF infants did not possess these differences in the gut microbiome according to colonization status. Thus, breastfed infants colonized with *C. difficile* appear to possess a gut microbiome that differs from non-colonized infants and resembles that of EFF infants, but the driving force and direction of this association remains unknown. Understanding these compositional differences as drivers of *C. difficile* colonization may be important to ensure future childhood health.

## Introduction

*Clostridioides* (formerly *Clostridium*) *difficile* is a bacterium that is present in the intestine of nearly 40% of infants at 1 month of age, and 30% of infants between the ages of 1 and 6 month (1). *C. difficile* is the main cause of antibiotic-associated diarrhea in adults (2, 3) and although *C. difficile* may not be accompanied by diarrheal illness in infants, it has been associated with atopy and microbial dysbiosis (4–6). Furthermore, despite the lack of immediate risks related to carriage of *C. difficile* in infants, this gram-negative spore-forming bacterium is capable of inducing gut inflammation and disrupting the intestinal epithelial barrier (7, 8). As a result, these less than desirable influences on the intestinal environment may impact the succession and abundance of commensal gut microbiota and overall microbial ecology.

Infancy is a critical period for establishment of the gut microbial ecosystem and immune system priming to confer protection against gut microbial dysbiosis and reduce the risk of negative health outcomes. *C. difficile* is thought to promote colonization of non-commensals and pathogenic bacteria, although this phenomenon has received little attention in infants. In a small group of infants (*n* = 53) (6), one study found that *Ruminococcus gnavus* and *Klebsiella pneumoniae* species were more prevalent in infants colonized with *C. difficile*, while non-carriers were more frequently colonized by *Bifidobacterium longum*. Acquisition of *C. difficile* during infancy has been attributed to several environmental exposures, notably formula feeding (1, 9, 10). Breastmilk bioactive factors, including human milk oligosaccharides and secretory Immunoglobulin A (sIgA), neutralize toxins and bind pathogens, which may account for asymptomatic colonization of the infant gut with *C. difficile* and/or lower colonization rates in breastfed infants vs. infants not fed human milk (11–13). Consequently, infants colonized with *C. difficile* may manifest distinct and persistent changes in their gut ecology, including changes in metabolites, secretory proteins and resident microbiota. Hence, the relationship between *C. difficile* and the infant gut microbiome merits further examination.

In this study, we report the association between *C. difficile* (family Peptostreptococcaceae) and other gut microbiome components, including composition, metabolites and sIgA, to provide insights into ecological factors related to *C. difficile* expansion in infancy. We also explored these differences in exclusively breastfed, partially breastfed, and exclusively formula fed infants to examine the gut microbial community and *C. difficile* colonization infants with distinct diets.

## Methods

### Study Design and Population

This study includes a sub-set of 1,562 families enrolled in the CHILD Cohort Study. In this prospective population-based cohort, mothers were recruited and enrolled with informed consent during the second or third trimester of pregnancy between January 2009 and December 2012 from the Vancouver, Edmonton and Manitoba study sites (inclusion and exclusion criteria outlined at www.childstudy.ca) (14). The primary objective of the CHILD Cohort Study was to determine the developmental, environmental, and genetic determinants of later allergy and asthma in childhood (15). All infants included in this subsample provided a fecal sample at 3–4 months of age, which was sequenced by Illumina MiSeq and processed by targeted qPCR to detect *C. difficile*. Within this study, smaller, yet representative, groups of samples were profiled to describe concentrations of fecal metabolites (*n* = 467) and secretory IgA (*n* = 731) (Supplementary Table 1). Gut microbiota compositional findings have previously been described for infants in the CHILD Cohort Study (16), but this paper is the first integration and report of 4 characterizations of the infant gut microbiome and gut immunity from the CHILD Cohort Study. The Human Research Ethics Boards at the University of Manitoba, University of Alberta, and University of British Columbia approved this study.

### qPCR for *Clostridioides difficile* Detection

Fecal samples of 5–10 grams were collected from infant diapers during home-visits conducted at 3–4 months of age by a research assistant or parents according to an approved protocol (Supplementary Figure 1). Samples were aliquoted and stored at −80°C until analyzed. A targeted 16S primer and probe set was used for amplification and quantification of *C. difficile* and followed the methods set by Penders et al. (17). To minimize differential inhibitory effects due to variable concentrations of genomic template DNA in qPCR, all template DNA samples were first normalized by dilution to 1 ng/μL (18). Then, each multiplex assay was prepared to contain 1X QuantiNova Multiplex PCR Kit (QIAGEN), 0.4 μM of each primer, 0.25 μM of each probe and 1 μL [1 ng/μL] of sample DNA in a final volume of 20 μL. qPCR cycling conditions were as follows: initial denaturation for 2 min at 95.0°C, 40 cycles of denaturation for 5 s at 95°C and annealing/extension/reading for 20 s at 60°C. Oligonucleotides were acquired from IDT (Integrated DNA Technologies Inc, Coralville, IA, USA) and reactions were performed on the MiniOpticonTM Real-Time PCR System (Bio-Rad, Hercules, CA, USA). A standard curve was created and employed to determine the efficiency of the *C. difficile* primers and probes by performing five 1:10 serial dilutions of *C. difficile* ATCC 9689D-5 genomic DNA starting at 1 ng/μL. We calculated the lower limit of detection for the multiplex assay to be 1X10-5 ng of DNA or 2 genomes of *C. difficile* based on the amplification data from the serial dilution and the non-template control. Because each template sample represented a different starting mass of stool, the limit of quantification for the analysis was variable from sample-to-sample, and ranged from 514 to 33,333 genomes/g stool. Infants were classified by *C. difficile* colonization status (present in fecal sample, yes/no). Amongst colonized infants, median levels of *C. difficile* (ng/g feces) in infant fecal samples were not different between feeding groups (data not shown).

### Fecal Microbiome Analysis

DNA extraction and amplification of bacterial V4 hypervariable region of the bacterial 16S rRNA gene was followed by sequencing and taxonomic classification and was conducted as previously described (19). To summarize, microbial DNA was extracted from the frozen stool samples mentioned above (80 to 200 mg) using the QIAamp DNA Stool Mini kit according to the manufacturer protocol (Qiagen Inc, Valencia CA). Next, the bacterial 16S rRNA genes were amplified at the hypervariable V4 region using PCR with appropriate primers. PCR products were combined for sequencing, performed using the Illumina MiSeq platform (San Diego, CA). Resultant sequences were taxonomically classified and matched at >97% similarity against the Greengenes reference database in QIIME and filtered/excluded if <60% similarity. Finally, microbiota data were rarefied to 13,000 sequences per sample and relative abundances were calculated. At this time, microbiota diversity within samples (alpha diversity) was calculated using standardized estimators of OTU richness and/or evenness: Chao1 and Shannon diversity indices.

#### Short-Chain Fatty Acid (SCFA) and Other Fecal Metabolites

In a sub-set of fecal samples (*N* = 467), metabolites were quantified by magnetic resonance spectroscopy (NMR). NMR requires a small quantity of sample for processing and has high reproducibility compared to mass spectrometry (20). Homogenization of 100 mg of sample and subsequent centrifugation were performed as necessary for sample cleaning: Each sample was placed in an Eppendorf tube will 1 mL of ice water, vortexed for 5 min and subjected to sonication for 20 more minutes at 4°C. Samples were then vortexed for another 20 min at 250 rpm. Samples were then centrifuged at 15,000 × g for 1 h at 4°C. The supernatant was removed and placed in a new tube and the process was repeated. The cleaned fecal water was stored at −20°C. After extraction, 280 μL of fecal water was mixed with 70 μL of a standard buffer solution (54% D_2_O: 46% 750 mM potassium phosphate (mono- and dibasic) pH 7.0 v/v containing 5 mM DSS-d_6_ (2,2-dimethyl-2-silcepentane-5-sulphonate). The sample (350 μL) was then transferred to 3 mm SampleJet NMR tube for subsequent spectral analysis. All ^1^H-NMR spectra were collected on a 700 MHz Avance III (Bruker) spectrometer equipped with a 5 mm HCN Z-gradient pulsed-field gradient (PFG) cryoprobe. ^1^H-NMR spectra were acquired at 25°C using the first transient of the NOESY pre-saturation pulse sequence (noesy1dpr), chosen for its high degree of quantitative accuracy.

Prior to spectral analysis, all FIDs (free induction decays) were zero-filled to 250 K data points and line broadened 0.5 Hz. The methyl singlet produced by a known quantity of DSS was used as an internal standard for chemical shift referencing (set to 0 ppm) and for quantification. All ^1^H-NMR spectra were processed and analyzed using the Chenomx NMR Suite Professional software package version 8.1 (Chenomx Inc., Edmonton, AB) (11). The Chenomx NMR Suite software allows for qualitative and quantitative analysis of an NMR spectrum by manually fitting spectral signatures from an internal database to the spectrum. Typically 90% of visible peaks were assigned to a compound and more than 90% of the spectral area could be routinely fit using the Chenomx spectral analysis software. Most of the visible peaks are annotated with a compound name. We sought to identify all metabolites relevant to microbial production or substrate use. Metabolites were quantified as μmol/gram feces. In this study, we report on a subset of metabolites measured, specifically the SCFAs acetate, butyrate, and propionate, in addition to other metabolites in the metabolic pathways of *C. difficile* including para-cresol, succinate, and glutamate (Supplementary Figure 2).

#### Fecal Secretory IgA

A sub-sample of fecal samples were assayed for sIgA (*N* = 731) using the Secretory IgA ELISA (enzyme-linked immunosorbent assay) kit (ELISA, Immundiagnostik AG assay, Bensheim, Germany). Approximately 14 mg of fecal sample was used for the sIgA analyses. Samples were run in duplicate according to the manufacturer's protocol, as previously described (21), and quantified as the average milligram of sIgA per gram wet weight feces (mg/g). To summarize, a fecal sample aliquot for each infant was thawed and an IDK Extract buffer was used to extract fecal sIgA. Samples were then diluted (1:125) with a wash buffer and placed in a microtiter plate along with controls and standards. Wells were aspirated, washed and 100 μL of conjugate was added and allowed to incubate at room temperature. Samples were then shaken on a horizontal mixer, washed with TMB substrate and incubated in the dark (20 min). An ELISA reader was used to measure the absorption at 450 nm (620 nm reference). The reads were multiplied by 12,500 and compared against a standard curve, created using standards provided with the assay kit, for quantification.

### Covariate Data

Breastfeeding status was determined through self-report questionnaires administered to mothers at 3–4 months postpartum (*N* = 1,554). A 3-category variable was created for infant breastfeeding status at the time of stool sample collection and questionnaire administration: (1) exclusively breastfed (EBF), (2) partially (i.e., mixed) breastfed (PBF), and (3) exclusively formula fed (EFF). Complete feeding data were missing in 8 infants, leaving a total of 1554 infants (not the full *N* = 1,562 with available *C. difficile* and microbiome data) that were stratified by feeding mode.

### Statistical Analysis

All statistical analysis was conducted using Stata (version 13), RStudio (version 1.1.456), and the Galaxy platform (MaAslin) between September 2018 and March 2019. Non-parametric (Mann-Whitney U or Kruskal–Wallis test) and parametric (student's *t*-test) tests were used where appropriate (Supplementary Figure 3) to compare alpha diversity indices, fecal metabolites, and fecal sIgA according to colonization status. Differences in taxon relative abundance (outcomes) according to *C. difficile* colonization status (predictor) were determined using the multivariate association with linear models method developed by the Huttenhower lab (MaAslin) (22) (available at: https://huttenhower.sph.harvard.edu/galaxy/). Spearman correlations were computed in Supplementary Table 2, and heatplots were generated using the gplots package and the heatmap.2() command in R. Scatter bar graphs were generated using the ggplots2 package and the geom_boxplot() and geom_beeswarm() commands. Statistical significance was defined as a two-sided *p* or *q*-value < 0.05, after FDR correction for multiple comparisons.

## Results and Discussion

**Figure 1:**
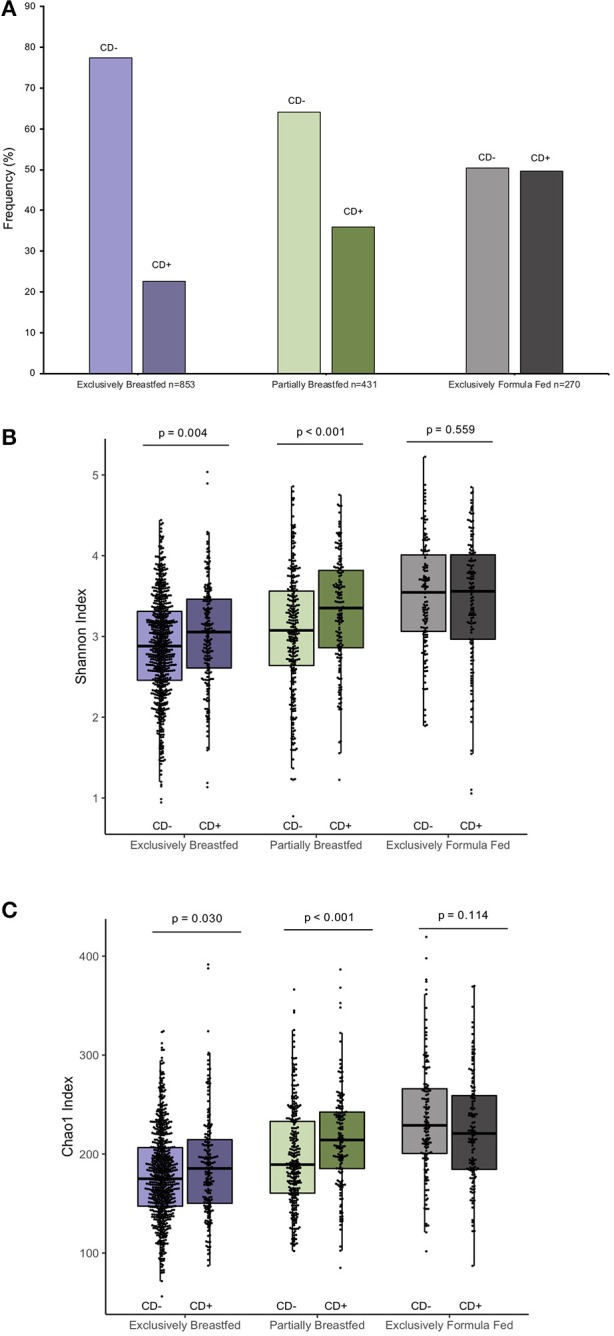
Frequency of *C. difficile* colonization in our study population and infant microbial alpha-diversity according feeding mode (*n* = 1,554). Colonization rates differ within feeding groups **(A)** 22.63% of exclusively breastfed infants (*N* = 193/853), 35.96% of partially breastfed infants (*N* = 155/431) and 49.63% of formula fed infants were colonized (*N* = 134/270) (Fishers' exact *p* < 0.001). Scatter box-plots of the median (middle line), Q3 and Q1 quartiles (box limits), IQR (whiskers) and outlying values (dots). Data were normally distributed (Supplementary Figure 3) and thus two-sided *p*-values were calculated with students *t*-test within infant feeding groups, comparing colonized and non-colonized infants at a significance threshold of α = 0.05. Higher α-diversity was observed for infants colonized with *C. difficile* (CD+) and breastfed (either exclusively or partially) than non-carriers (CD–) on the same diet. This was the case for both the Shannon diversity index **(B)** and Chao1 species richness index **(C)**. Purple represents EBF, green for PBF and gray for EFF.

The prevalence of *C. difficile* colonization among all study infants was 30.9% (*n* = 482/1562), which aligns with previously reported estimates (1). These colonization rates differed between feeding groups: 22.6% for EBF, 36.0% for PBF and 49.6% for EFF infants (χ^2^: 76.71, *p* < 0.001, Figure 1A, *N* = 1,554). The mean Shannon and Chao1 indices for EBF and PBF infants were lower for infants who lacked *C. difficile* compared to infants colonized with *C. difficile*, suggesting that the richness and abundance of the infant gut microbiota are greater and more equally distributed in the presence of *C. difficile* (p < 0.05, Figures 1B,C). No differences in alpha diversity were detected with *C. difficile* colonization in EFF infants. These differences across feeding modality could not be attributed to the normal progression of microbiota development since infant age [median (IQR)] in each of the feeding groups was similar: 3.29 months (1.03) for EBF, 3.33 months (0.94) for PBF, and 3.20 months (1.10) for EFF, *p* = 0.27.

**Figure 2:**
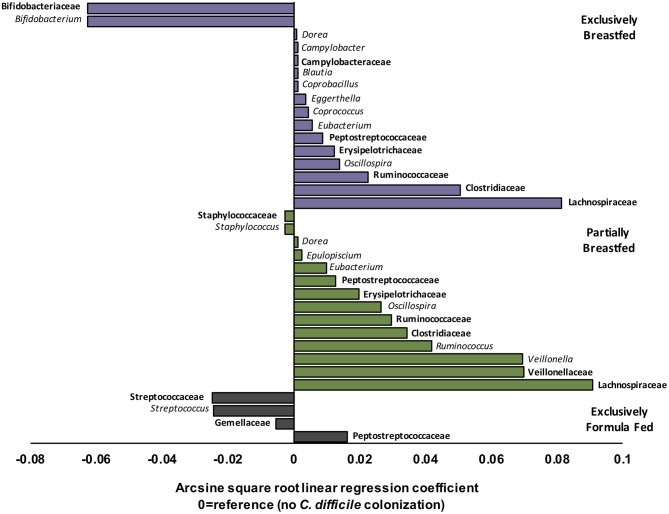
Relative differences in microbiota composition between *C. difficile* carriers and non-carriers across infant feeding groups (*n* = 1,554). Multivariate linear regression results (MaAslin) for family (**bolded**) and genus level taxa that are differentially associated with *C. difficile* colonization at 3–4 months of age. Values on the x-axis represent arcsine square root transformed regression coefficients of microbiota relative abundances for each linear model, adjusted for multiple comparisons (FDR correction) to determine which taxa are uniquely associated with *C. difficile* colonization. Each model had a reference of infants without *C. difficile* colonization at 3–4 months. Data shown only for taxa with FDR corrected two-sided *q*-value < 0.05. Coefficients > 0 (positive values) represent taxa that enriched in *C. difficile* carriers, while coefficients < 0 (negative values) represent taxa that were depleted in *C. difficile* carriers. *P*-values for each regression can be found in Supplementary Tables 3–5. Purple represents EBF (*N* = 853, 193 CD+), green for PBF (*N* = 431, 155 CD+) and gray for EFF (*N* = 270, 134 CD+).

EBF is generally associated with low microbial alpha diversity due to the dominance of *Bifidobacterium* spp. (19, 23). Bifidobacteria thrive on human milk oligosaccharides but their growth is reported to be suppressed with *C. difficile* colonization (6, 24). Accordingly, our regression models revealed that *Bifidobacterium* spp. were less abundant in EBF infants colonized with *C. difficile* than EBF infants who were not colonized (transformed β = −0.06, *q* = 0.021, Figure 2). Bifidobacteria are well-known acetate producers (24, 25) and their presence was positively correlated with this metabolite (*R* = 0.56, *p* < 0.01, Supplementary Figure 4). Despite an observed lowered relative abundance of *Bifidobacterium*, we measured higher absolute concentrations of fecal acetate among EBF infants colonized with *C. difficile* (*p* = 0.01, Supplementary Figure 2). Many other microbiota produce acetate (26); thus, the greater diversity of microbes we observed in EBF *C. difficile* positive infants likely contributed to higher fecal acetate levels. In our study, acetate concentrations were also positively correlated with the members of the Campylobacteraceae (*R* = 0.38, *p* > 0.10), Peptostreptococcaceae (*R* = 0.55, *p* = 0.05) and Clostridiaceae (*R* = 0.58, *p* > 0.10) families (Supplementary Figure 4) which were enriched in EBF infants positive for *C. difficile* (*q* < 0.05, Figure 2).

Other microbes that were differentially abundant in the presence of *C. difficile* were members of the Lachnospiraceae and the Ruminococcaceae families, and both were enriched with *C. difficile* colonization among EBF and PBF infants (*q* < 0.05, Figure 2). Among EBF infants, we also observed higher absolute concentrations of non-acetate SCFAs (i.e., butyrate and propionate, *p* < 0.05, Supplementary Figure 2) when they were colonized with *C. difficile*. The relative abundance of Ruminococcaceae [e.g., *Oscillospira* spp. which are butyrate producers (27)] was positively correlated with butyrate (*R* = 0.35, *p* < 0.01, Supplementary Figure 4) and with p-cresol (*R* = 0.27, *p* = 0.08, Supplementary Figure 4), a known product of *C. difficile* amino acid metabolism (28). The fecal concentrations of p-cresol were higher in all infants colonized with *C. difficile*, regardless of infant feeding group (*p* < 0.01, Supplementary Figure 2). Lachnospiraceae was weakly correlated with propionate concentrations (*R* = 0.18, *p* < 0.01). Propionate production by Lachnospiraceae is through the 1,2-propanediol and acrylate pathways, which are possessed by *Blautia, Eubacterium*, and *Coprococcus* (29), all genera that were enriched in EBF *C. difficile* carriers (*q* < 0.05 for each, Figure 2).

Correlations between microbial relative abundance and butyrate concentrations involved a greater number of gut microbiota in PBF than EBF infants colonized with *C. difficile*. Specifically, Ruminococcaceae (R: 0.25, *p* < 0.01), Lachnospiraceae (R: 0.28, *p* < 0.01) and Clostridiaceae (R: 0.46, *p* < 0.01) were all positively correlated with butyrate and enriched in PBF infants (*q* < 0.05 for each, Figure 2). In contrast to EBF infants, Lachnospiraceae taxa in PBF *C. difficile* positive infants were inversely correlated with propionate levels (*R* = −0.57, *p* < 0.01). Since Bacteroidaceae are more abundant with any formula feeding (16), irrespective of *C. difficile* status in the current study, and they predominantly produce propionate (26), these microbiota likely out-competed Lachnospiraceae in the fermentation of substrates in PBF infants to produce propionate via the succinate pathway. Consistently, we observed a positive correlation between propionate concentrations and relative abundance of Bacteroidaceae among PBF and EFF infants, which was absent in EBF infants and independent of *C. difficile* status (Supplementary Figure 4).

Unique to PBF infants colonized with *C. difficile* was a higher relative abundance of *Veillonella* spp. (family Veillonellaceae, *q* = 0.002, Figure 2). Also, the relative abundance of *Staphylococcus* spp. (family *Staphylococcaceae, q* < 0.001, Figure 2) was lower in PBF infants positive for *C. difficile* than non-carriers. Fewer compositional differences were detected with *C. difficile* colonization among EFF infants, relative to breastfed (exclusive and partial) infants and equally no differences were detected in fecal metabolites. The sole family of microbes whose relative abundance was significantly higher in EFF *C. difficile* carriers was its own family, the Peptostreptococcaceae (*q* = 4.80E−24, Figure 2). As also expected, the Peptostreptococcaceae family were enriched in EBF and PBF infants colonized with *C. difficile* (*q* < 0.001, Figure 2).

Other metabolites measured in our study include glutamate and succinate. Glutamate, a metabolite shown to play a role in the establishment of *C. difficile in vivo* (30), was not differentially associated with *C. difficile* colonization in any of the feeding groups (Supplementary Figure 2). This metabolite is essential for *C. difficile* pathogenesis but may not be required for asymptomatic colonization in infants. Further, unlike glutamate dehydrogenase, a protein marker of *C. difficile* colonization (30), glutamate is an intermediary metabolite which may be consumed in several microbiota cross-feeding pathways. In fact, fecal levels of glutamate correlated with key microbes that differed by *C. difficile* status in all feeding groups (Supplementary Figure 4). Similarly, *C. difficile* utilizes succinate for its expansion and has the ability to ferment succinate to butyrate (31). Consistent with the succinate pathway, succinate concentrations were lower and concentrations of butyrate higher with *C. difficile* colonization in EBF infants and PBF infants (*p* = 0.05, Supplementary Figure 2). Since succinate is not easily absorbed by colonic cells (32), as suggested by our findings, levels may be further lowered from cross-feeding by succinate-utilizing members of the “Negativicutes” branch of Firmicutes clade (e.g. *Veillonella* spp.) (32, 33). Indeed, succinate was negatively correlated with Veillonellaceae in PBF infants (Supplementary Figure 4).

**Figure 3:**
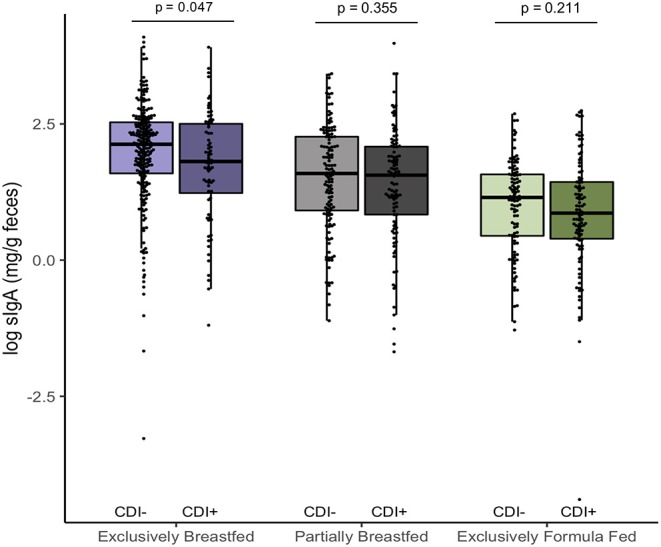
Log transformed measures of fecal secretory IgA, according to infant colonization and feeding mode (*n* = 731). Scatter box-plots of the median (middle line), Q3 and Q1 quartiles (box limits), IQR (whiskers) and outlying values (dots). Two-sided *p*-values were calculated with Mann–Whitney *U*-test of log transformed fecal sIgA (mg/g) comparing colonized and non-colonized infants within the same diet group. Exclusively breastfed infants colonized with *C. difficile* (CD+) had lower median fecal sIgA than non-carriers (CD–) on the same diet. Purple represents EBF (*N* = 290, 72 CD+), green for PBF (*N* = 237, 104 CD+) and gray for EFF (*N* = 204, 101 CD+).

In addition to examining fecal metabolites, we also measured fecal sIgA levels as a marker of intestinal homeostasis and mucosal immunity (34). As we previously reported, *C. difficile* was associated with lower sIgA concentrations among EBF infants (*p* = 0.047, Figure 3) (11). Since infant secretion of sIgA has been positively correlated with breastmilk sIgA levels and breastmilk microbiota, maternal factors may contribute to lower concentrations in the infant (35, 36). Notably, animal models have shown that offspring nursed by mothers who are sIgA-deficient have a different gut microbiota composition than those receiving sIgA through breastmilk (37, 38). Similar to what we observed in EBF *C. difficile* positive infants, reduced fecal sIgA was associated with compositional differences that included an increased relative abundance of Lachnospiraceae and pro-inflammatory microbiota. Previous work from the CHILD Cohort Study has shown that sIgA in breastmilk may be depleted due to factors such as depression (21) or an altered maternal milk microbiota (36), which may predispose the infant to colonization by *C. difficile* and related dysbiosis. Although sIgA can bind enteric pathogens (34), there is a lack of evidence suggesting that *C. difficile* contributes to the destruction of sIgA or reduce production of this protein.

Finally, some of our findings suggest that the gut microbiota of breastfed (both EBF and PBF) infants colonized with *C. difficile* resembles the gut microbial composition of adults (e.g., increased relative abundance of Firmicutes such as *Eubacterium* spp.) (39). Meta-analytic evidence from cohorts worldwide documents similarity between the gut microbiota of EFF infants and that of adults (23). Extending this evidence, our study suggests that the gut microbiome of breastfed infants colonized with *C. difficile* is compositionally similar to that observed in EFF infants (Supplementary Figure 5).

In our large population cohort study, we were not able to categorize infants according to the proportional intake of breastmilk vs. formula, as others have (40). Since our study did not employ culture-based methodology, another study limitation was inability to detect the strains and toxigenic properties of *C. difficile*. Should our study findings continue to align with previous findings, we might expect a prevalence of toxigenic strains to be <10% among infants with *C. difficile* positive samples (12, 41). We are also unable to determine the direction of observed associations: whether *C. difficile* caused gut microbial dysbiosis, or whether gut dysbiosis increased infant susceptibility to *C. difficile* colonization. This could be improved by measuring the *C. difficile* colonization status of infants longitudinally (at more than one time point) to assess if *C. difficile* colonization is transient or persistent and whether the microbiome changes precede or follow colonization. However, with enhanced characterization of the gut microbiome beyond taxon composition, our study provides evidence for a putative role of *C. difficile* colonization on the gut microbial ecology of young, full-term infants from a large, general population in North America.

## Conclusion

We observed a distinct gut microbiome in young infants colonized with *C. difficile* and this distinction depended on the breastfeeding status of the infant. The most noticeable microbiome differences with *C. difficile* colonization, especially depletion of *Bifidobacterium* spp., were among EBF infants. Similar compositional differences among members of the Firmicutes phylum were seen in EBF and PBF infants. However, unique to PBF infants was enrichment of Veillonellaceae. These findings highlight the differential relationship of *C. difficile* colonization on EBF vs. PBF vs. EFF infants, which should be considered in future studies of infants feeding modality and disease risk. In summary, we found differences in the infant gut microbiome with *C. difficile* colonization, but it remains unclear whether *C. difficile* causes these differences or if external factors in early infancy create a niche that is more permissive to colonization. Newer cohorts with available multi-omics data could validate these findings and explore the hypothesized relations between various microbiota and *C. difficile* to further understand colonization of this microbe in infancy and its implications in later childhood health.

## Data Availability Statement

The data and analysis code that support the findings of this study can be made available from the corresponding author and CHILD Cohort Study coordinators upon reasonable request. These data, including study participant data, are securely stored in the CHILDdb.ca database.

## Ethics Statement

The studies involving human participants were reviewed and approved by the University of Alberta, University of British Columbia and University of Manitoba Ethics Boards. Written informed consent to participate in this study was provided by the participants' legal guardian/next of kin.

## Author Contributions

KD and AK conceived the study. KD performed data analysis, prepared figures, and drafted and edited the manuscript. HT generated gut microbiota operational taxonomic unit profiles using QIIME software. TK conducted DNA extraction and sample preparation for sequencing for microbiome analyses. NM-L performed targeted qPCR for *C. difficile* detection. DW and RM supervised and conducted NMR and fecal metabolite analyses. CF supervised, conducted, and helped interpret the fecal sIgA analyses. MA created the breastfeeding measures. DG, AB, PM, PS, ST, TM, MS, DL, and JS obtained funding and contributed to study design and data collection. AK obtained funding, contributed to data interpretation and critically reviewed the manuscript. All the authors reviewed the manuscript content, provided feedback and approved the final version. AK will serve as guarantor of the manuscript's contents.

### Conflict of Interest

The authors declare that the research was conducted in the absence of any commercial or financial relationships that could be construed as a potential conflict of interest.
