# Supplementary material for: Clostridioides difficile Colonization Is Differentially Associated With Gut Microbiome Profiles by Infant Feeding Modality at 3–4 Months of Age
Source: Front Immunol. 2019 Dec 11;10:2866. doi: 10.3389/fimmu.2019.02866 (PMC6917614; doi:10.3389/fimmu.2019.02866)

Supplementary Material

Supplementary Table 1| Distribution of population characteristics according to feeding modality and analysis sub-samples.

	Exclusively breastfed			Partially breastfed			Exclusively formula fed		
	Full sample (N=853)	Metabolite sample (N=178)	sIgA sample (N=290)	Full sample (N=431)	Metabolite sample (N=165)	sIgA sample (N=237)	Full sample (N=270)	Metabolite sample (N=124)	sIgA sample (N=204)
**N (%)**									
**Birth mode**									
Vaginal no IAP	472 (56.59)	108 (62.79)	169 (59.30)	219 (52.52)	74 (46.54)	114 (49.78)	142 (53.99)	67 (54.92)	108 (54.27)
Vaginal IAP	200 (23.98)	35 (20.35)	60 (21.05)	89 (21.34)	36 (22.64)	55 (24.02)	54 (20.53)	23 (18.85)	40 (20.10)
Elective CS	57 (6.83)	7 (4.07)	23 (8.07)	53 (12.71)	23 (14.47)	27 (11.79)	29 (11.03)	15 (12.30)	20 (10.05)
Emergency CS	105 (12.59)	22 (12.79)	33 (11.58)	56 (13.43)	26 (16.35)	33 (14.41)	38 (14.45)	17 (13.93)	31 (15.58)
**Hospitalization since birth**									
No	787 (93.69)	167 (95.43)	272 (95.77)	393 (92.47)	150 (92.59)	217 (92.34)	247 (93.21)	116 (95.08)	188 (94.47)
Yes	53 (6.31)	8 (4.57)	12 (4.23)	32 (7.53)	12 (7.41)	18 (7.66)	18 (6.79)	6 (4.92)	11 (5.53)
**Infant antibiotics**									
No	826 (96.83)	173 (97.19)	281 (96.90)	417 (96.75)	159 (96.36)	228 (96.20)	263 (97.41)	122 (98.39)	132 (98.51)
Yes	27 (3.17)	5 (2.81)	9 (3.10)	14 (3.25)	6 (3.64)	9 (3.80)	7 (2.59)	2 (1.61)	2 (1.49)
**Furry pets in the home**									
No	483 (57.43)	107 (60.80)	169 (59.91)	213 (50.35)	80 (49.69)	116 (49.57)	137 (51.31)	64 (52.03)	95 (47.26)
Yes	358 (42.57)	69 (39.20)	115 (40.49)	210 (49.65)	81 (50.31)	118 (50.43)	130 (48.69)	59 (47.97)	106 (52.74)
**Infant gender**									
Female	409 (47.97)	84 (47.19)	138 (47.59)	409 (47.95)	72 (43.64)	104 (43.88)	133 (49.26)	66 (53.23)	97 (47.55)
Male	444 (52.05)	94 (52.81)	152 (52.41)	444 (52.05)	93 (56.36)	133 (56.12)	137 (50.74)	58 (46.77)	107 (52.45)

***Birth mode and infant gender obtained from enrolled study participants birth charts and medical records**

****Hospitalization, antibiotic use and furry pet ownership were self-reported by families enrolled in the study in questionnaires administered at the 3-month study follow-up.**

Supplementary Table 2| Spearman correlations between microbiota and fecal metabolites among different infant feeding groups according to *Clostridioides difficile* colonization status. Spearman rho calculated using Stata (version 13.0). *attached excel spreadsheet with multiple tabs due to the large nature of this table.

Supplementary Table 3| Relative differences in microbiota composition between *C. difficile* carriers and non-carriers among EBF infants (n=853). Multivariate linear regression results (MaAslin) for family and genus level taxa that are differentially associated with *C. difficile* colonization at 3 months. Coeff. are arcsine square root transformed regression coefficients of microbiota relative abundances for each linear model, adjusted for multiple comparisons (FDR correction) to determine which taxa are uniquely associated with *C. difficile* colonization. Each model had a reference of infants without *C. difficile* colonization at 3-months. Data shown only for taxa with FDR corrected two-sided q-value < 0.05. Coefficients > 0 (positive values) represent taxa that enriched in *C. difficile* carriers, while coefficients < 0 (negative values) represent taxa that were depleted in *C. difficile* carriers.

**Colonized with *C. difficile* (ref: not colonized)**	*Coeff*	*N*	*Non-zero N*	*p-value*	*q-value*
Firmicutes|Clostridia|Clostridiales|Peptostreptococcaceae	0.009	853	203	0.000	0.000
Firmicutes|Clostridia|Clostridiales|Lachnospiraceae	0.081	853	800	0.000	0.000
Coprococcus	0.004	853	90	0.000	0.000
Dorea	0.001	853	152	0.002	0.011
Blautia	0.001	853	227	0.000	0.004
Lachnospira	0.008	853	78	0.001	0.008
Firmicutes|Clostridia|Clostridiales|Ruminococcaceae	0.023	853	540	0.000	0.000
Oscillospira	0.014	853	357	0.000	0.000
Firmicutes|Erysipelotrichi|Erysipelotrichales|Erysipelotrichaceae	0.012	853	467	0.001	0.005
Coprobacillus	0.001	853	25	0.000	0.002
Eubacterium	0.006	853	171	0.000	0.002
Proteobacteria|Epsilonproteobacteria|Campylobacterales|Campylobacteraceae	0.001	853	164	0.001	0.006
Campylobacter	0.001	853	164	0.001	0.006
Actinobacteria|Coriobacteriia|Coriobacteriales|Coriobacteriaceae *Eggerthella*	0.004	853	354	0.001	0.008
Firmicutes|Clostridia|Clostridiales|Clostridiaceae	0.051	853	742	0.002	0.010
Actinobacteria|Actinobacteria|Bifidobacteriales|Bifidobacteriaceae	-0.063	853	832	0.004	0.021
Bifidobacterium	-0.063	853	831	0.004	0.021

Supplementary Table 4| Relative differences in microbiota composition between *C. difficile* carriers and non-carriers among PBF infants (n=431). Multivariate linear regression results (MaAslin) for family and genus level taxa that are differentially associated with *C. difficile* colonization at 3 months. Coeff. are arcsine square root transformed regression coefficients of microbiota relative abundances for each linear model, adjusted for multiple comparisons (FDR correction) to determine which taxa are uniquely associated with *C. difficile* colonization. Each model had a reference of infants without *C. difficile* colonization at 3-months. Data shown only for taxa with FDR corrected two-sided q-value < 0.05. Coefficients > 0 (positive values) represent taxa that enriched in *C. difficile* carriers, while coefficients < 0 (negative values) represent taxa that were depleted in *C. difficile* carriers.

**Colonized with *C. difficile* (ref: not colonized)**	*Coeff.*	*N*	*Non-zero N*	*p.value*	*q.value*
Firmicutes|Clostridia|Clostridiales|Peptostreptococcaceae	0.013	431	135	3.87E-60	3.46E-58
Firmicutes|Erysipelotrichi|Erysipelotrichales|Erysipelotrichaceae	0.020	431	293	3.63E-06	9.29E-05
Eubacterium	0.010	431	146	6.27E-08	2.80E-06
Firmicutes|Clostridia|Clostridiales|Lachnospiraceae	0.091	431	408	8.71E-06	0.0002
Ruminococcus	0.042	431	312	2.91E-05	0.0004
Epulopiscium	0.002	431	133	6.21E-05	0.0008
Dorea	0.001	431	140	0.007	0.044
Firmicutes|Clostridia|Clostridiales|Clostridiaceae	0.034	431	393	1.91E-05	0.0003
Firmicutes|Bacilli|Bacillales|Staphylococcaceae	-0.003	431	121	1.94E-05	0.0003
Firmicutes|Bacilli|Bacillales|Staphylococcaceae|Staphylococcus	-0.003	431	121	1.94E-05	0.0003
Firmicutes|Clostridia|Clostridiales|Ruminococcaceae	0.030	431	346	0.0008	0.007
Oscillospira	0.026	431	256	0.0002	0.003
Firmicutes|Clostridia|Clostridiales|Veillonellaceae	0.070	431	429	0.0005	0.004
Veillonella	0.069	431	424	0.0003	0.003

Supplementary Table 5| Relative differences in microbiota composition between *C. difficile* carriers and non-carriers among EFF infants (n=270). Multivariate linear regression results (MaAslin) for family and genus level taxa that are differentially associated with *C. difficile* colonization at 3 months. Coeff. are arcsine square root transformed regression coefficients of microbiota relative abundances for each linear model, adjusted for multiple comparisons (FDR correction) to determine which taxa are uniquely associated with *C. difficile* colonization. Each model had a reference of infants without *C. difficile* colonization at 3-months. Data shown only for taxa with FDR corrected two-sided q-value < 0.05. Coefficients > 0 (positive values) represent taxa that enriched in *C. difficile* carriers, while coefficients < 0 (negative values) represent taxa that were depleted in *C. difficile* carriers.

**Colonized with *C. difficile* (ref: not colonized)**	*Coeff.*	*N*	*Non-zero N*	*p.value*	*q.value*
Firmicutes|Clostridia|Clostridiales|Peptostreptococcaceae	0.016	270	137	5.24E-26	4.80E-24
Firmicutes|Bacilli|Lactobacillales|Streptococcaceae	-0.025	270	270	0.0003	0.005
Streptococcus	-0.025	270	270	0.0002	0.005
Firmicutes|Bacilli|Gemellales|Gemellaceae	-0.006	270	166	4.58E-05	0.001

Supplementary Figure 1| Three-month stool sample collection protocol and form.

CHILD Study

ID

Event Name:

Sample Full Name: Child's Stool

Sample Short Name: STOOL

Sample ID:

Research Staff

Sample Collected: ◯ Yes ◯ No

Sample Collection Date and Time: Y M D T : (24 hr clock)

Weight: mg

Notes (e.g. if not collected or protocol deviation):

Instructions

Collection:

1. Label a stool specimen jar with the mother's name, CHILD study ID, and HealthDiary Specimen label.
2. The day before the visit, ask the mother to save the first poopy diaper and place in fridge in bag provided, writing the time of collection on the label.
3. Wearing nitrile gloves, take the poopy diaper into the bathroom, and collect stool to fill up to one half of the pre-labeled specimen jar.
4. Discard diaper as per the mother's instructions.
5. Ensure cap is screwed on tightly.
6. Place in cooler for transport to the Study centre.
7. On this form, record the time and date the child pooped.

Processing:

1. Freeze at -80 degrees Celsius.
2. Enter volume and collection information in HealthDiary.

CHILD Study Information

Canadian Healthy Infant Longitudinal Development Study

Toronto: 416-586-4800 X2977 Winnipeg: 204-789-3978 Edmonton: 780-407-8084 Vancouver: 604-875-2345 X5370

Coordinating Study Centre 905-522-1155 X35228

www.CanadianChildStudy.ca © HealthDiary Inc F37SPECIMEN v.2008M12D15

Supplementary Figure 2| Differences in the concentration of infant fecal metabolites, according to infant colonization and feeding mode (n=467). Scatter box-plots of the median (middle line), Q3 and Q1 quartiles (box limits), IQR (whiskers) and outlying values (dots). Two-sided p-values were calculated with Mann-Whitney U-test of the absolute concentrations of relevant fecal metabolites (µmol/g feces) comparing colonized and non-colonized infants within the same diet group. Concentrations of fecal metabolites (**a**= acetate, **b**=butyrate, **c**=propionate, **d**=glutamate, **e**=succinate, **f**=p-cresol) were differentially associated with *C. difficile* colonization and infant diet. (CD+ = colonized with *C. difficile*, CD- = non-carriers). Purple represents EBF (N=178, 47 CD+), green for PBF (N=165, 71 CD+) and grey for EFF (N=124, 65 CD+).

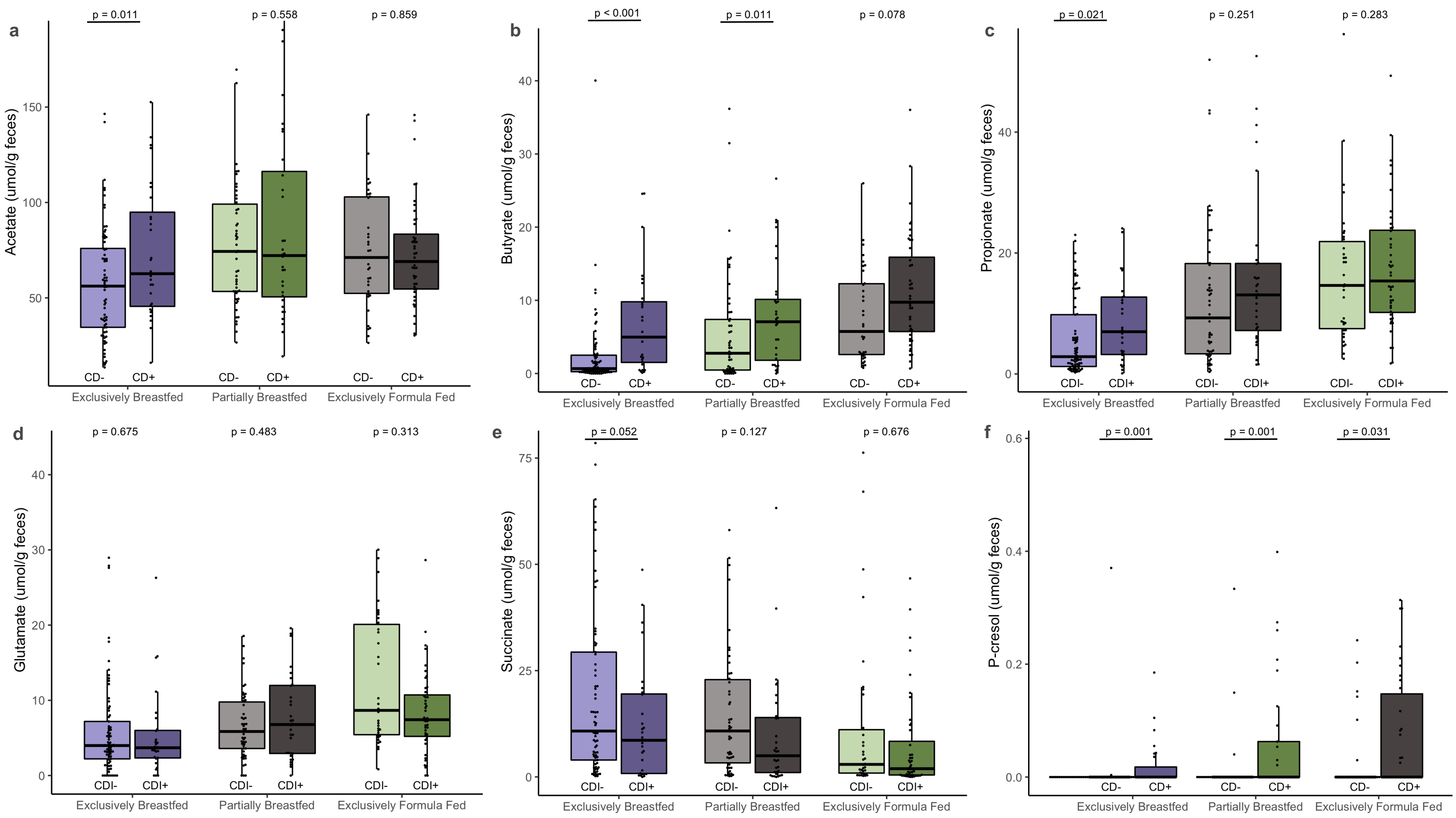
a
p = 0.011
p = 0.558
p = 0.859
Acetate (umol/g feces)
CD-
CD+
Exclusively Breastfed
Partially Breastfed
Exclusively Formula Fed
b
p < 0.001
p = 0.011
p = 0.078
Butyrate (umol/g feces)
c
p = 0.021
p = 0.251
p = 0.283
Propionate (umol/g feces)
CDI-
CDI+
d
p = 0.675
p = 0.483
p = 0.313
Glutamate (umol/g feces)
e
p = 0.052
p = 0.127
p = 0.676
Succinate (umol/g feces)
f
p = 0.001
p = 0.001
p = 0.031
P-cresol (umol/g feces)

Supplementary Figure 3| Histogram of the distribution of alpha diversity indices among study participants. Chao1 (**a**) and Shannon (**b**) alpha diversity indices were normally distributed in our sub-sample of 1562 infants. This allowed us to use t-tests to assess the relationship between these indices and *C. difficile* colonization.

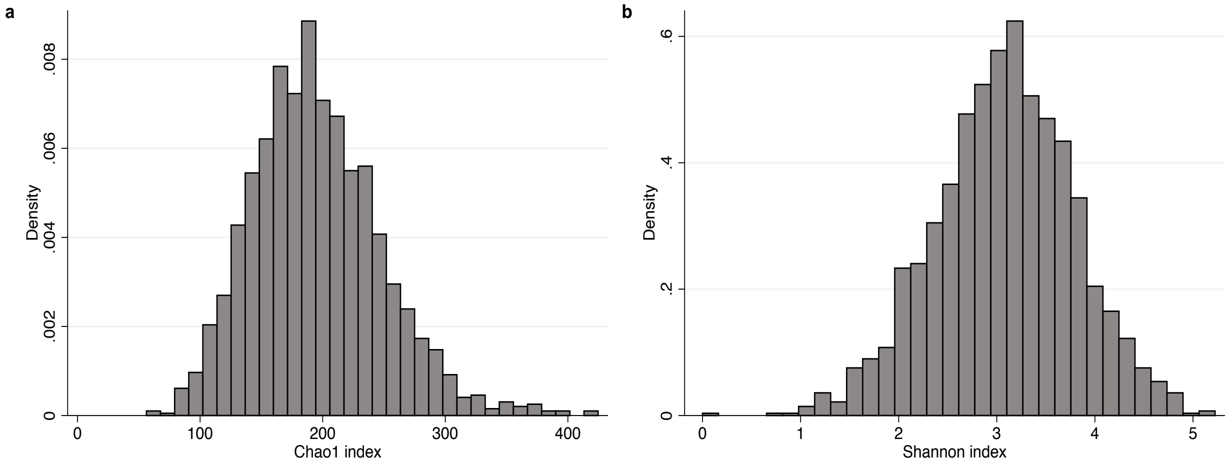

Supplementary Figure 4| Spearman correlation heatmaps depicting the relationships between fecal metabolites, sIgA and infant gut microbiota (n=467). Colors indicate the Spearman rho (R) values with red values approaching a value of negative 1, white or light values approaching a value of zero and blue values approaching a value of positive 1. All exact Spearman rho coefficients and corresponding p-values can be found in Supplementary Table 5. The top 10 most abundant taxa are represented at the family level on the y-axis. Correlations were used to describe relationships between our other quantitative findings and were assessed for statistical significance. Weak or negligible correlation: ≤0.29, low to moderate correlation: 0.30-0.49, moderate to strong correlation: ≥0.50. Purple represents EBF (N=178, 47 CD+), green for PBF (N=165, 71 CD+) and grey for EFF (N=124, 65 CD+).

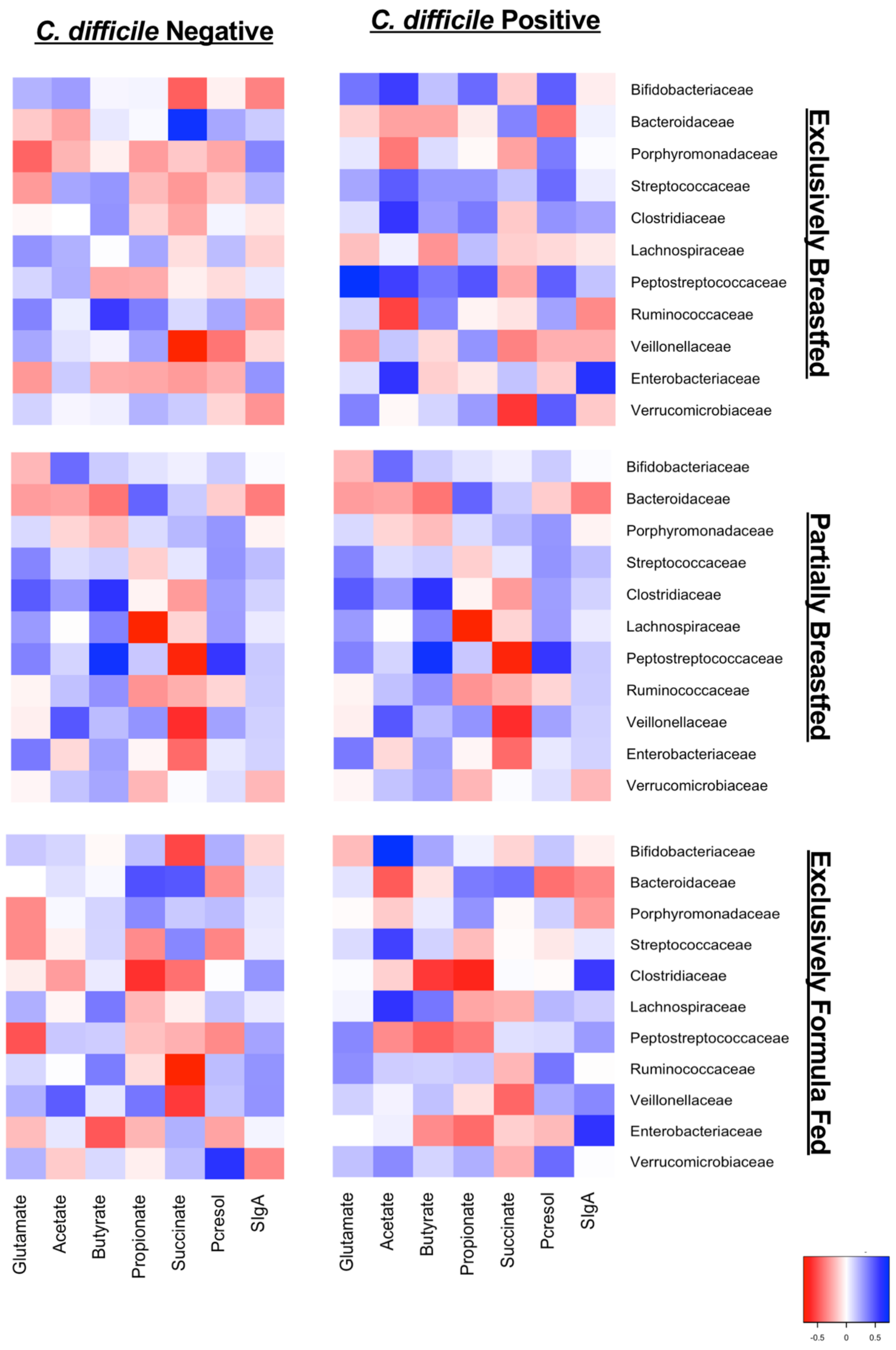

C. difficile Negative
C. difficile Positive
Exclusively Breastfed
Partially Breastfed
Exclusively Formula Fed
Bifidobacteriaceae
Bacteroidaceae
Porphyromonadaceae
Streptococcaceae
Clostridiaceae
Lachnospiraceae
Peptostreptococcaceae
Ruminococcaceae
Veillonellaceae
Enterobacteriaceae
Verrucomicrobiaceae
Glutamate
Acetate
Butyrate
Propionate
Succinate
Pcresol
SIgA
-0.5
0
0.5

Supplementary Figure 5| Relative abundance stacked bar plots depicting differences in composition of gut microbiota according to *C. difficile* colonization status and feeding mode (N=1554). Descriptive depiction of the compositional microbiome profile, at the family level, for the study infants. Please see Figure 2 in the main manuscript for more a more detailed analysis of microbiota composition.

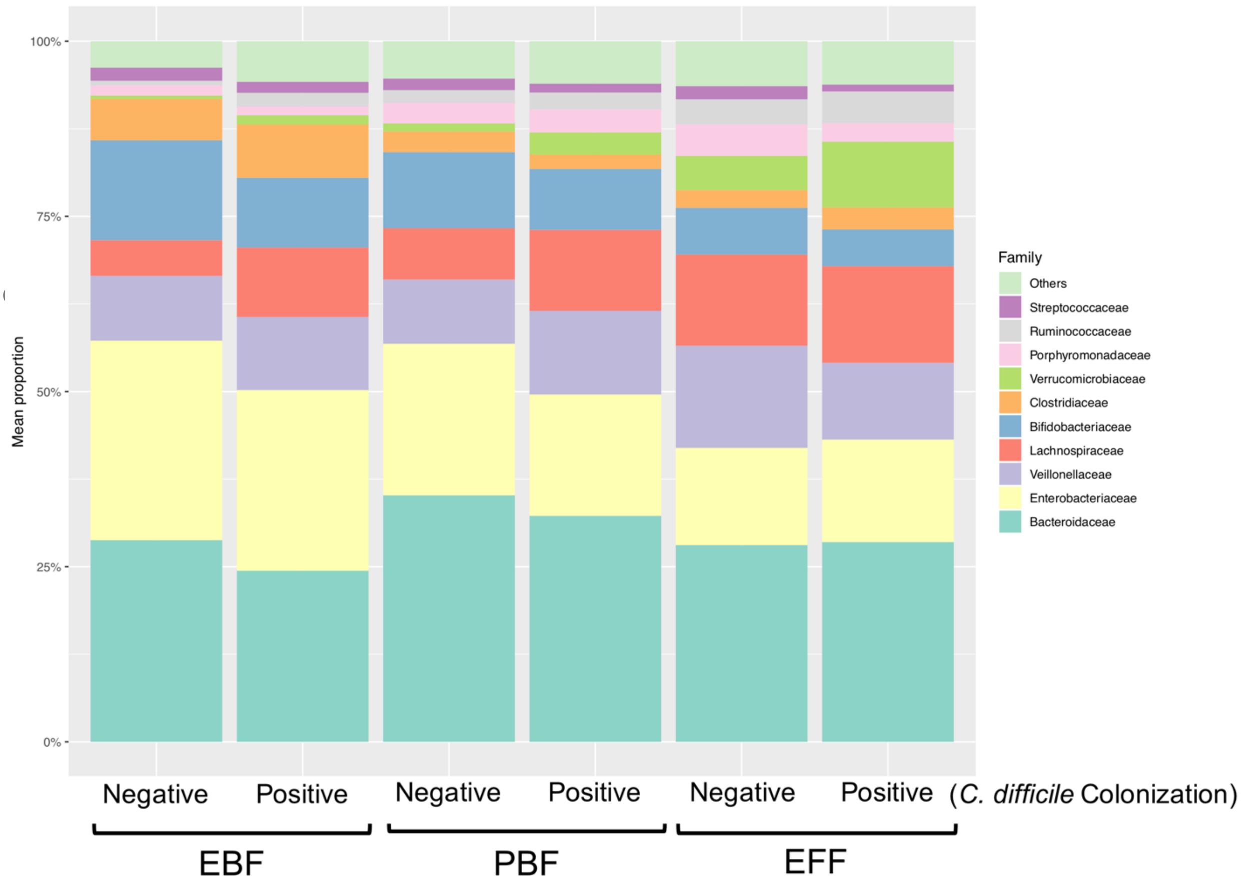

Supplement: Supplementary file 1 [file Table_1.pdf]
